# Th2 Cytokines IL-4, IL-13, and IL-10 Promote Differentiation of Pro-Lymphatic Progenitors Derived from Bone Marrow Myeloid Precursors

**DOI:** 10.1089/scd.2022.0004

**Published:** 2022-06-08

**Authors:** Maria Espinosa Gonzalez, Lisa Volk-Draper, Nihit Bhattarai, Andrew Wilber, Sophia Ran

**Affiliations:** ^1^ Department of Medical Microbiology, Immunology, and Cell Biology, Southern Illinois University School of Medicine, Springfield, Illinois, USA.; ^2^ Simmons Cancer Institute, Southern Illinois University School of Medicine, Springfield, Illinois, USA.

**Keywords:** lymphatic endothelial progenitors, lymphangiogenesis, Th2 cytokines, myeloid cell differentiation

## Abstract

Myeloid-lymphatic endothelial cell progenitors (M-LECP) are a subset of bone marrow (BM)-derived cells characterized by expression of M2-type macrophage markers. We previously showed significant contribution of M-LECP to tumor lymphatic formation and metastasis in human clinical breast tumors and corresponding mouse models. Since M2 type is induced in macrophages by immunosuppressive Th2 cytokines IL-4, IL-13, and IL-10, we hypothesized that these factors might promote pro-lymphatic specification of M-LECP during their differentiation from BM myeloid precursors. To test this hypothesis, we analyzed expression of Th2 cytokines and their receptors in mouse BM cells under conditions leading to M-LECP differentiation, namely, CSF-1 treatment followed by activation of TLR4. We found that under these conditions, all three Th2 receptors were strongly upregulated in >95% of the cells that also secrete endogenous IL-10, but not IL-4 or IL-13 ligands. However, addition of any of the Th2 factors to CSF-1 primed cells significantly increased generation of myeloid-lymphatic progenitors as indicated by co-induction of lymphatic-specific (eg, Lyve-1, integrin-a9, collectin-12, and stabilin-1) and M2-type markers (eg, CD163, CD204, CD206, and PD-L1). Antibody-mediated blockade of either IL-10 receptor (IL-10R) or IL-10 ligand significantly reduced both immunosuppressive and lymphatic phenotypes. Moreover, tumor-recruited Lyve-1^+^ lymphatic progenitors in vivo expressed all Th2 receptors as well as corresponding ligands, including IL-4 and IL-13, which were absent in BM cells. This study presents original evidence for the significant role of Th2 cytokines in co-development of immunosuppressive and lymphatic phenotypes in tumor-recruited M2-type myeloid cells. Progenitor-mediated increase in lymphatic vessels can enhance immunosuppression by physical removal of stimulatory immune cells. Thus, targeting Th2 pathways might simultaneously relieve immunosuppression and inhibit differentiation of pro-lymphatic progenitors that ultimately promote tumor spread.

## Introduction

The lymphatic system plays a paramount role in absorption of fluid and proteins, regulation of immunity, and tissue repair [1,2]. Expansion of lymphatic vessels (LV), that is, lymphangiogenesis, is significantly increased in tumors [3] and chronically inflamed sites [4]. Tumor-induced lymphangiogenesis promotes metastasis to lymph nodes (LNs) from which malignant cells spread to distant organs, the process primarily responsible for patient mortality [3,5]. The key event regulating lymphangiogenesis is activation of vascular endothelial growth factor receptor-3 (VEGFR-3) expressed in lymphatic endothelial cells (LEC) by its ligands VEGF-C/-D [6,7].

We previously showed that lymphangiogenesis is also regulated by bone marrow (BM)-derived myeloid-lymphatic endothelial cell progenitors (M-LECP) that express VEGFR-3 and other LEC markers [8–10]. Recruitment of M-LECP to murine and human tumors strongly correlates with increased LV density and metastasis to LNs [10,11]. It is therefore of clinical interest to identify M-LECP in tumors and delineate the mechanisms responsible for their differentiation.

Prior characterization of M-LECP showed the following: (1) they are derived from BM myeloid precursors induced by CSF-1 [9,10], the main promoter of myeloid-macrophage lineage [12]; (2) acquisition of lymphatic phenotype in CSF-1-primed myeloid precursors is induced by TLR4 pathway activation [9]; (3) they are identified by a unique signature of co-expressed stem, myeloid, and LEC markers [9,10]; and (4) tumor-recruited M-LECP can be classified as tumor-associated macrophages (TAMs) as both populations share specific markers of immunosuppressive M2 type. Although classification to stimulatory M1 and immunosuppressive M2 does not account for all functional states of macrophages, TAMs typically express CD163 [13], CD204 [14], and CD209 [15] surface proteins. These markers are highly upregulated in M-LECP recruited to clinical breast cancers as was determined by their expression in TLR4^+^/CD11b^+^ TAMs, which were also positive for lymphatic markers Lyve-1, podoplanin (Pdpn), or Vegfr-3 [10].

The main functions of M2 macrophages are resolution of inflammation [16] and regeneration of injured tissues [17], including vascular remodeling [18]. Chronic inflammatory conditions, such as in tumor microenvironment (TME), induce the M2-type to quell excessive immune stimulation and trigger tumor repair. The switch from M1 to M2 phenotype is primarily induced by Th2 cytokines IL-4 [19], IL-13 [20], and IL-10 [21]. These cytokines expressed in many human cancers [22] suppress antitumor immunity [23,24], promote blood and LV formation [25,26], and increase metastasis [27–29].

CSF-1-primed BM cells treated with Th2 cytokines generate M2-type myeloid cells resembling TAMs [30,31]. IL-4, which shares its pathway with IL-13 [32], is known to induce Lyve-1 and other LEC markers in tumor-recruited CD11b^+^ cells [33], whereas deficiency in IL-10 receptor (IL-10R) caused impaired lymphatic formation due to decreased generation of M2 macrophages [34]. Taken together with M2 marker expression of M-LECP, these studies suggest that Th2 cytokines play a role in generation of lymphatic progenitors in the BM.

To test this hypothesis, we compared differentiation of BM cells using either a standardized CSF-1/TLR4 protocol [9] or Th2 cytokines applied after CSF-1 priming. We then determined basal and induced expression of Th2 cytokines and their receptors. We found that all Th2 receptors and IL-10 were highly upregulated during M-LECP differentiation induced by TLR4 ligand in CSF-1-primed cells. In contrast to BM, tumors expressed IL-10, IL-4, and IL-13, thus providing a conducive environment for activation of all three Th2 pathways in receptor-positive M-LECP. This study presents original evidence for induction of pro-lymphatic differentiation by immunosuppressive Th2 factors, which underscores an intimate link between immunosuppression and lymphangiogenesis that jointly promote metastasis.

## Materials and Methods

### Antibodies and cytokines

All primary antibodies used for flow cytometry and immunofluorescence, as well as IL-10 and IL-10R blockade are listed in Supplementary Table S1. All secondary antibodies were purchased from Jackson ImmunoResearch (West Grove, PA). Recombinant mouse CSF-1, IL-4, IL-13, and IL-10 were purchased from BioLegend (San Diego, CA).

### Ethics statement

Animal experiments were conducted in accordance with recommendations in the Guide for the Care and Use of Laboratory Animals of the National Institute of Health. Protocols were approved by the Laboratory Animal Care and Use Committee of Southern Illinois University School of Medicine (Protocols 187-13-021 and 187-20-004).

### M-LECP differentiation from BM cells

BM cells isolated from the long bones of C57BL/6 mice were crushed in 10 mL of PBS containing 0.5% BSA and 2 mM EDTA. After passaging the suspension through a 70-μm strainer, cells were spun down at 1,000 rpm for 10 min. Cell pellets were resuspended in 5 mL of growth medium (DMEM containing 10% FBS and standard supplements) and counted.

Approximately 10 × 10^6^ cells in 10 mL of growth medium with CSF-1 (10 ng/mL) were seeded in 10 cm^2^ dishes coated with 10 μg/mL of fibronectin. After 3 days, attached cells were washed with DPBS and stimulated with CSF-1 combined with IL-4, IL-13, IL-10 (10 ng/mL each), or LPS (3 nM) until day 6. Some experiments were performed in the presence of anti-IL-10 or anti-IL-10R blocking antibodies or rat control IgG. On day 6, cells were imaged, counted, and analyzed by flow cytometry or extracted of total RNA for RT-qPCR.

### Flow cytometry

Flow cytometry analysis was performed using 1 × 10^5^ cells/sample. All incubations were performed on ice. Cells were preincubated with mouse gamma globulins (10 μg/mL) for 10 min to block Fc receptor. This was followed by a 1-h incubation with 5 μg/mL of a primary antibody, washing with F-buffer (2% BSA and 0.2% of sodium azide in DPBS), and incubation with 1 μg/mL of appropriate secondary antibody. Stained cells were fixed for 10 min with 1% paraformaldehyde followed by washing and resuspension in 250 μL of F-buffer. Targets were detected by AccuriC6 flow cytometer (BD Biosciences) and analyzed using FlowJo software (Tree Star, Ashland, OR). Target expression was quantified in duplicate with three biological replicates. Results are presented as the mean percentage of positive cells and mean fluorescent intensity (MFI) ± SD.

### Enzyme-linked immunosorbent assay

Conditioned medium (CM) from BM cells was collected on days 3 and 6 of differentiation. Lysates from MMTV-PyMT and EMT6 tumors were prepared 4–6 days postimplantation. IL-4 and IL-13 were quantified using kits from Peprotech (East Windsor, NJ). IL-10 was quantified by a kit from R&D Systems (Minneapolis, MN). Cytokine concentrations were calculated based on the standard curve generated for purified standards supplied with commercial kits. Concentrations in CM and tumor lysates are expressed as pg/mL and pg/mg of total protein, respectively.

### Primer design and validation

Primers were designed from CDS of mouse targets in the NCBI database (Bethesda, MD). Sequences with unique specificity to target genes were selected using GeneRunner software and online NCBI Primer BLAST alignment tool (http://blast.ncbi.nlm.nih.gov/blast.cgi). Primers purchased from Integrated DNA Technologies (Coralville, IA) were validated by qPCR using mouse universal cDNA as a template. The quality of each primer was confirmed by a single peak on melting curve analysis and amplification efficiency of Ct slope regression for four cDNA dilutions with *R*^2^ ≥ 0.95 being acceptable. Only qPCR-validated primers that produced a single band of correct size in bp as visualized on a 2% agarose gel were used. All primer sequences are shown in Supplementary Table S2.

### RNA isolation and RT-qPCR

RNA extraction and cDNA synthesis were performed using RNeasy Mini and SuperScript VILO cDNA synthesis kits, respectively, according to the manufactures' instructions (Thermo Fisher, Rockford, IL). Concentrations and quality of RNA and cDNA were determined by NanoDrop2000. Triplicate samples containing primers listed in Supplementary Table S2 were mixed with GoTaq Master Mix (Promega, Madison, WI) and analyzed by Master-Cycle Realplex PCR machine (Eppendorf, NY). Reaction conditions consisted of an initial denaturation step at 95°C for 1 min followed by 38 cycles of denaturation, annealing at 95°C, and extension at 60°C. A final melting curve was calculated by heating from 60°C to 90°C. Data were normalized by cDNA concentration and relative mRNA expression was determined using the ΔΔCt method.

### Immunofluorescence

MMTV-PyMT and EMT6 mouse breast carcinoma lines were orthotopically implanted into C57BL/6 and BALB/c female mice, respectively. Snap-frozen tumors of 500 mm^3^ were cut into 8-μm-thick sections and fixed for 10 min with acetone. Sections were rehydrated in PBS supplemented with 0.1% Tween-20 (PBST) before incubation with Image-iT FX signal enhancer (Thermo Fisher) for 30 min at RT. Primary antibodies were diluted 1:100 in PBST containing 0.2% BSA and incubated with tissues overnight at 4°C. Incubation with secondary antibodies diluted 1:100 in the same buffer was for 1 h at 37°C. Slides were counter stained with Hoechst stain (2 μg/mL), fixed with 1% paraformaldehyde, and mounted in Prolong Gold medium (Thermo Fisher). Slides were washed between each step in PBST for 10 min. Images were acquired on an Olympus BX41 microscope equipped with a DP70 digital camera and DP Controller software (Olympus, Tokyo, Japan).

### Statistical analysis

Statistical analysis was assessed using GraphPad Prism software (La Jolla, CA). All results were expressed as mean ± SD. Student's two-tailed *t*-test was used for comparative analyses. The *P* value ≤0.05 was considered statistically significant.

## Results

### TLR4 activation of CSF-1-primed myeloid precursors upregulates Th2 receptors and IL-10

**FIG. 1.**
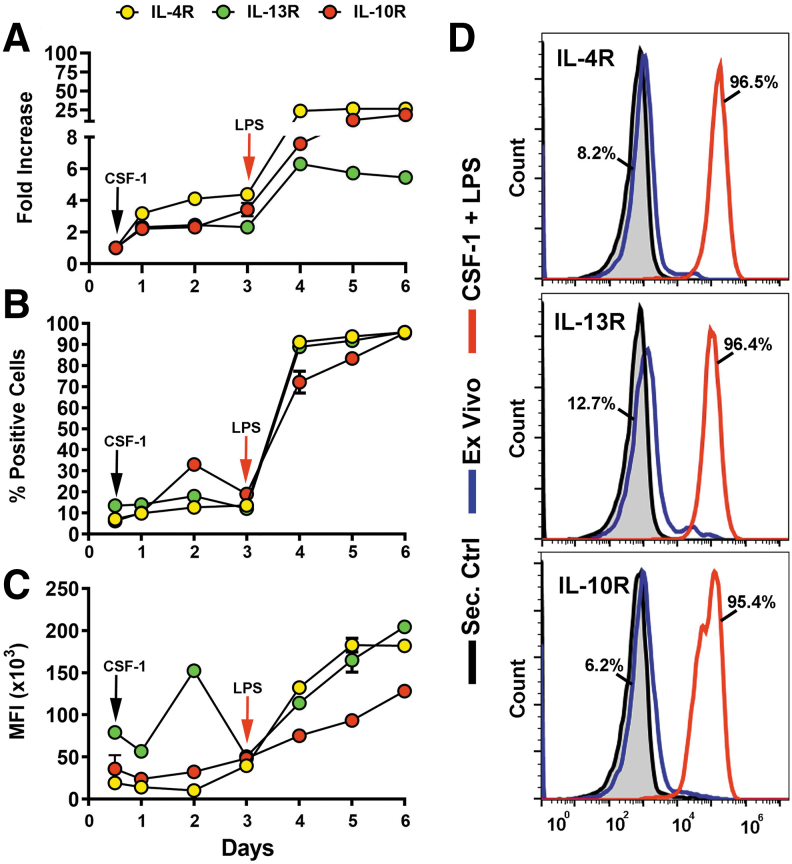
Upregulation of IL-4R, IL-13R, and IL-10R during CSF-1/LPS induced differentiation of M-LECP. Mouse BM cells were differentiated with CSF-1 followed by LPS as described under Materials and Methods section. Transcripts and protein levels of IL-4R, IL-13R, and IL-10R were determined daily by qPCR **(A)** and flow cytometry **(B–D)**, respectively. Quantitative PCR data in **(A)** are presented by fold increase of target transcripts in differentiated M-LECP compared with ex vivo cells. Values represent the mean ± SD of triplicate or duplicate for qPCR and flow cytometry analyses, respectively. **(B, C)** Mean percent of positive cells and MFI for each target were determined daily. Note that some SD bars are not visible due to their smaller size compared with the symbol. **(D)** Representative histograms of IL-4R, IL-13R, and IL-10R protein expression in ex vivo (*blue lines*) and differentiated (*red lines*) cells. The *black line* represents cells stained with secondary antibody alone. Percent of positive ex vivo and differentiated cells are indicated for each target. All analyses were reproduced three times. BM, bone marrow; LPS, lipopolysaccharide; M-LECP, myeloid-lymphatic endothelial cell progenitor; MFI, mean fluorescent intensity.

**FIG. 2.**
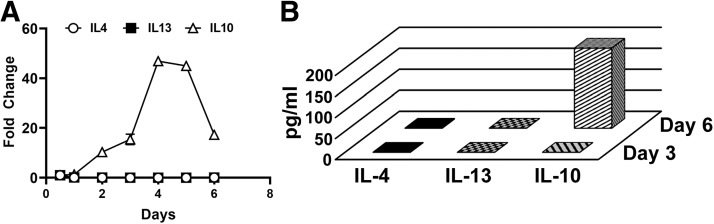
Expression of IL-4, IL-13, and IL-10 cytokines in BM cells differentiating into M-LECP. BM cells were differentiated with CSF-1 followed by LPS as described under Materials and Methods section. **(A)** Fold change in mRNA expression of the indicated cytokines was calculated based on cDNA concentration-normalized Ct values for qPCR. **(B)** Conditioned medium of differentiating BM cells collected on days 3 and 6 was analyzed for secreted IL-4, IL-13, and IL-10 proteins using ELISA. All values represent mean ± SD of duplicate of experiments that were reproduced three times. ELISA, enzyme-linked immunosorbent assay.

We previously established that treatment of CSF-1-preconditioned BM cells with TLR4 ligands generates myeloid-lymphatic progenitors [9]. We also showed that human tumor-recruited M-LECP express specific markers of M2 macrophages [10]. These findings suggested that Th2 cytokines IL-4, IL-13, and IL-10, which control generation of M2 macrophages [30], might promote differentiation of M-LECP. To test this hypothesis, we determined the expression of Th2 receptors and their ligands during differentiation of CSF-1/TLR4-activated BM cells. We found that CSF-1 and a TLR4 ligand lipopolysaccharide (LPS) upregulated Th2 receptors by fourfold and 25-fold, respectively, compared with ex vivo cells (Fig. 1A–C). On day 6, > 95% of cells were positive for all three Th2 receptors by flow cytometry (Fig. 1D). In contrast, only *IL10*, but not *IL4* or *Il13*, was detected among transcripts and CM of M-LECP determined by qPCR and enzyme-linked immunosorbent assay, respectively (Fig. 2).

### CSF-1-primed BM cells respond to exogenous Th2 cytokines by upregulating their receptors that confer the immunosuppressive phenotype

Although exogenous Th2 cytokines alone failed to sustain survival of BM cells (Supplementary Table S3), when added to CSF-1-primed cells, they did support cell survival and upregulated matched receptors.

**FIG. 3.**
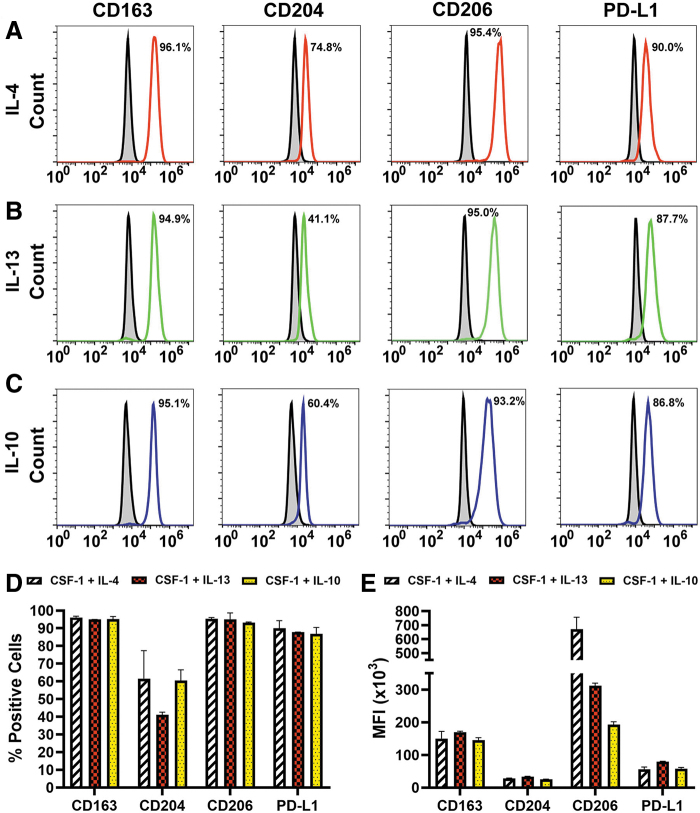
Th2 cytokines upregulate immunosuppressive M2 markers in BM myeloid precursors. CSF-1-primed BM cells were stimulated with 10 ng/mL of IL-4 **(A)**, IL-13 **(B),** or IL-10 **(C)** instead of LPS. Expression of M2 surface markers CD163, CD204, CD206, and PD-L1 was determined by flow cytometry on day 6 of differentiation. Representative histograms of Th2 cytokine-induced M2 targets are presented. Numbers indicate percent of positive cells for each target. **(D)** Mean percent of positive cells ± SD and **(E)** mean MFI ( × 10^3^) ± SD for each M2 marker induced by IL-4, IL-13, or IL-10 in CSF-1-primed cells, as indicated by symbols. All analyses were reproduced three times.

Three lines of evidence indicate that the induced receptors were functional. First, cell densities and diameters were significantly increased after treatment with Th2 ligands compared with ex vivo and cells treated with CSF-1 alone (*P* ≤ 0.05, Supplementary Table S3 and Supplementary Figs. S1–S3). Second, all Th2 cytokines induced the immunosuppressive phenotype as demonstrated by substantial increase of M2-specific markers CD163, CD204, CD206, and PD-L1 from <10% in ex vivo to up to 96% in differentiated cells (Fig. 3). Induction of immunosuppressive phenotype is a well-known function of Th2 cytokines [35,36], and served here as a positive control for functionality of the receptors. Third, in line with known autocrine cross-regulation of Th2 pathways [37,38], all tested Th2 factors strongly induced corresponding receptors (Table 1).

**Table 1.** Effects of IL-4, IL-13, and IL-10 on Expression of Their Receptors in CSF-1-Primed Cells

Target Treatment	IL-4R	*P* value^a^	IL-13R	*P* value	IL-10R	*P* Value
Percent of positive cells^b^						
None (Ex vivo)^c^	7.10 ± 0.80	N/A	13.50 ± 0.40	N/A	6.20 ± 0.20	N/A
CSF-1	31.55 ± 3.00	N/A	60.15 ± 0.30	N/A	38.80 ± 7.63	N/A
IL-4	N/D	N/A	N/D	N/A	N/D	N/A
CSF-1 + IL-4	72.05 ± 1.55	<0.05	59.85 ± 1.34	0.4531	98.55 ± 0.21	<0.05
IL-13	N/D	N/A	N/D	N/A	N/D	N/A
CSF-1 + IL-13	86.63 ± 3.98	<0.05	75.17 ± 4.06	<0.05	89.15 ± 0.32	<0.05
IL-10	N/D	N/A	N/D	N/A	N/D	N/A
CSF-1 + IL-10	95.87 ± 2.67	<0.05	94.00 ± 2.53	<0.05	90.95 ± 1.85	<0.05
Mean fluorescent intensity ( × 10^3^)^b^						
None (Ex vivo)	19.30 ± 0.20	N/A	79.3 0 ± 3.50	N/A	35.99 ± 11.50	N/A
CSF-1	28.80 ± 1.94	N/A	56.71 ± 1.85	N/A	42.90 ± 0.88	N/A
IL-4	N/D	N/A	N/D	N/A	N/D	N/A
CSF-1 + IL-4	69.51 ± 16.68	<0.05	73.19 ± 2.66	<0.05	44.82 ± 2.76	<0.05
IL-13	N/D	N/A	N/D	N/A	N/D	N/A
CSF-1 + IL-13	105.41 ± 12.04	<0.05	87.46 ± 2.58	0.0980	63.81 ± 3.77	<0.05
IL-10	N/D	N/A	N/D	N/A	N/D	N/A
CSF-1 + IL-10	125.60 ± 11.25	<0.05	115.57 ± 9.95	<0.05	52.33 ± 1.90	<0.05

^a^ *P* values indicate significant differences compared with CSF-1 alone.

^b^ Percent of positive cells and mean fluorescent intensity values are presented as mean ± SD of three to five independent experiments performed in duplicate on day 6.

^c^ Ex vivo analysis was performed on the day of cell isolation.

N/A, not applicable; N/D, not done; analyses were not performed due to low cell survival.

The positivity for IL-4R, IL-13R, and IL-10R increased from 7% to 13% in naive cells to 30%–60% in CSF-1-treated cells, whereas subsequently added Th2 cytokines further increased the positive fraction up to 95% (*P* ≤ 0.05 for all cytokines, Table 1). Collectively, these data indicate that CSF-1-primed cells respond to Th2 cytokines by upregulating matching functional receptors that promote immunosuppressive phenotype.

### Th2 cytokines induce pro-lymphatic differentiation along with immunosuppressive phenotype

**FIG. 4.**
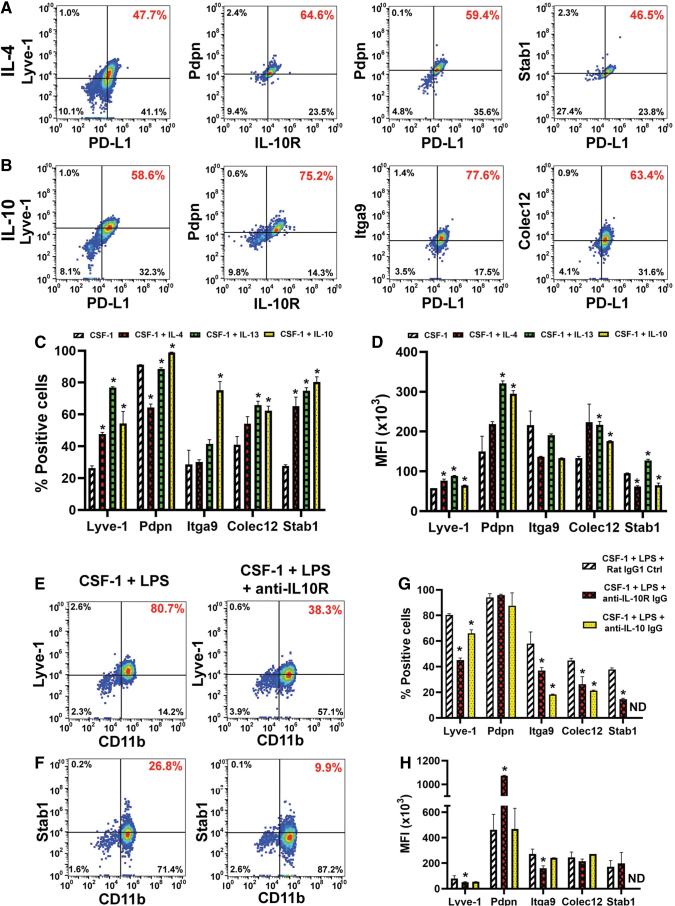
Th2 cytokines promote the lymphatic identity in CSF-1-primed myeloid precursors. BM cells were differentiated with CSF-1 and either **(A)** IL-4 or **(B)** IL-10. Flow cytometry *dot plots* of cells stained for M2 markers PD-L1 or IL-10R and lymphatic specific proteins Lyve-1, podoplanin (Pdpn), integrin-a9 (Itga9), collectin-12 (Colec12), or stabilin-1 (Stab1). Percentage of double-positive cells is highlighted in *red font*. **(C)** The mean percent of positive cells and **(D)** MFI ( × 10^3^) ± SD for each LEC target. Statistical significance between cells differentiated with Th2 cytokines and CSF-1 alone determined by a Student's *t*-test with *P* values ≤0.05 and it is indicated by *. **(E–H)** Standard differentiation of BM cells by CSF-1/LPS was performed in the presence of control or blocking antibodies to IL-10R or IL-10 ligand. On day 6, cells were stained for CD11b in combination with anti-Lyve-1 **(E)** or anti-stabilin-1 **(F)** antibodies. Percent of double-positive cells for each *dot plot* is identified in *red font*. **(G)** The mean percent of positive cells ± SD and **(H)** mean MFI ( × 10^3^) ± SD for each LEC marker expressed in control and IL-10 pathway blocking antibodies. Statistical significance between marker expression in differentiated cells in the presence of control and blocking antibodies was determined by a Student's *t*-test with *P* values ≤0.05 and it is indicated by *. ND (not done) indicates markers that were not analyzed in some assays. Each assay was performed in duplicate and reproduced in three independent experiments. LEC, lymphatic endothelial cell.

Once we established that induced receptors were functional, we examined the effects of Th2 factors on expression of lymphatic-specific markers that identify M-LECP [10,39]. CSF-1 alone increased the proportion of positive cells for four LEC markers Lyve-1 [40], stabilin-1 [41], integrin-a9 (Itga-9) [42], and collectin-12 [43] from 2% to 7% in the naive population to 26%–40% (Table 2 and Fig. 4A–D). Addition of IL-4, IL-13, or IL-10 increased this fraction up to 75%–96% for most LEC markers, except Pdpn. The latter is mainly regulated by CSF-1, although Th2 cytokines did increase its MFI by 3.3-fold (Fig. 4D and Table 2).

**Table 2.** Effects of IL-4, IL-13, and IL-10 on Lymphatic Endothelial Cell Marker Expression in CSF-1-Primed Cells

Target Treatment	Lyve-1	Pdpn	Stab1	Colec12	Itgα9
Percent of positive cells^a^					
None (Ex vivo)^b^	5.45 ± 0.15	6.03 ± 0.74	3.13 ± 0.26	2.23 ± 0.09	7.15 ± 0.15
CSF-1	26.20 ± 1.50	91.20 ± 0.10	27.60 ± 0.80	41.00 ± 5.10	28.50 ± 9.00
IL-4	N/D	N/D	N/D	N/D	N/D
CSF-1 + IL-4	47.60 ± 1.10^*^	64.30 ± 2.20	65.10 ± 5.80^*^	54.20 ± 4.40^*^	30.20 ± 1.30
IL-13	N/D	N/D	N/D	N/D	N/D
CSF-1 + IL-13	76.90 ± 0.50^*^	88.60 ± 0.80	74.80 ± 2.00^*^	65.90 ± 2.40^*^	41.50 ± 2.60^*^
IL-10	N/D	N/D	N/D	N/D	N/D
CSF-1 + IL-10	54.30 ± 7.60^*^	98.90 ± 0.20^*^	80.30 ± 3.30^*^	62.40 ± 2.80^*^	75.20 ± 5.40^*^
Mean fluorescent intensity ( × 10^3^)^a^					
None (Ex Vivo)	20.75 ± 1.55	36.83 ± 1.79	22.16 ± 1.42	26.86 ± 6.19	56.09 ± 1.54
CSF-1	57.60 ± 0.00	149.90 ± 38.40	94.70 ± 1.10	133.20 ± 4.20	216.20 ± 35.30
IL-4	N/D	N/D	N/D	N/D	N/D
CSF-1 + IL-4	76.60 ± 3.90^*^	218.80 ± 6.10	61.90 ± 2.00	223.00 ± 45.90	136.80 ± 1.10
IL-13	N/D	N/D	N/D	N/D	N/D
CSF-1 + IL-13	88.20 ± 1.20^*^	320.90 ± 6.40^*^	127.00 ± 3.40^*^	216.50 ± 8.80^*^	190.80 ± 2.90
IL-10	N/D	N/D	N/D	N/D	N/D
CSF-1 + IL-10	64.20 ± 1.30^*^	295.10 ± 7.80^*^	65.00 ± 5.40	175.70 ± 1.50^*^	133.00 ± 0.70

^a^ Percent of positive cells and mean fluorescent intensity values are presented as mean ± SD of three to five independent experiments performed in duplicate on day 6.

^b^ Analysis was performed on the day of cell isolation.

Asterisks indicate statistically significant increases compared with CSF-1 alone determined by Student's *t*-test.

N/D, not done; analyses were not performed due to poor cell survival.

The least induced marker was integrin-a9 (26%–37% positive cells after IL-4 or IL-13 treatment), whereas the most upregulated markers were Lyve-1 and stabilin-1 with twofold to fourfold increase in positive cells post-IL-10 treatment (Table 2). MFI for most markers was also significantly increased compared with CSF-1 alone (*P* ≤ 0.05, Table 2). Consistent with acquisition of the immunosuppressive phenotype (Fig. 3), LEC proteins were upregulated in cells that co-expressed M2 markers such as IL-10R and PD-L1 (Fig. 4A, B). This result strongly suggested that development of both phenotypes is co-regulated by Th2 stimuli.

Since IL-10 was endogenously induced by LPS, we tested whether specific blockade of this pathway could suppress acquisition of the lymphatic phenotype. Indeed, all LEC markers upregulated by IL-10 (Fig. 4A–D) were significantly reduced (*P* ≤ 0.05) by either anti-IL-10 or anti-IL-10R antibody (Fig. 4E–H). Consistent with data shown above, Pdpn was not affected, whereas stabilin-1 was the most sensitive to IL-10R inhibition as demonstrated by 3.7-fold decrease compared with control IgG. This is the first direct evidence that immunosuppressive cytokine IL-10 promotes differentiation of lymphatic endothelial progenitors.

### IL-10 inhibition switches M2 to M1 phenotype, which correlates with suppression of pro-endothelial and lymphatic-specific differentiation

**FIG. 5.**
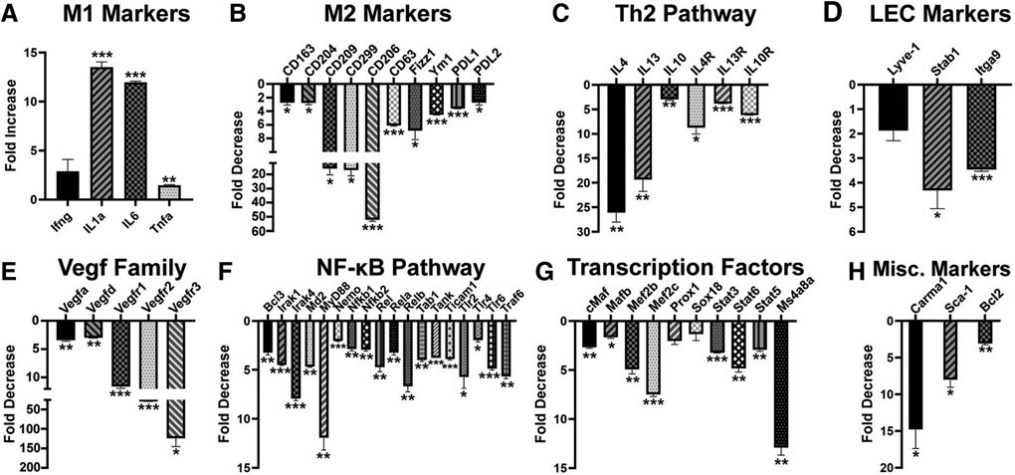
Blockade of IL-10 inhibits immunosuppressive M2 phenotype as well as pro-vascular and lymphatic endothelial differentiation. Total mRNA was isolated from BM cells differentiated with CSF-1 and LPS in the presence of a control and IL-10R blocking antibody. Relative expression of **(A)** M1 markers, **(B)** M2 markers, **(C)** Th2 pathway cytokines and receptors, **(D)** LEC markers, **(E)** Vegf family members, **(F)** NF-κB pathway regulators, **(G)** selected transcription factors, and **(H)** selected miscellaneous markers was determined by qPCR. The mean fold increase or decrease ± SD caused by an anti-IL-10R antibody relative to control IgG were calculated based on normalized Ct values. *P* values determined by Student's *t*-test are indicated by * ≤ 0.05, ** ≤ 0.01, and *** ≤ 0.001 for differences between expression levels in the presence of anti-IL-10R and control antibodies. All assays were performed in triplicate and reproduced twice.

We next characterized the impact of inhibiting the IL-10 pathway on differentiation of BM myeloid cells. Expression levels of M1, M2, Th2 regulators, endothelial, lymphatic, and other relevant markers were compared by qPCR in cells treated with anti-IL-10R or control antibody for the duration of differentiation. All analyzed M1 markers (*Il1α*, *Ifnγ*, *Il6*, and *Tnfα*) were upregulated, whereas all 10 analyzed M2 markers were significantly decreased by anti-IL-10R IgG (Fig. 5A, B). Consistently, transcripts of all Th2 cytokines and their receptors were significantly reduced by 5- to 25-fold (*P* ≤ 0.05, Fig. 5C). Likewise, transcripts of LEC markers decreased twofold to fourfold (*P* < 0.05, Fig. 5D).

Moreover, four members of pro-vascular VEGF family were reduced by 3- to 40-fold, whereas the transcript for key lymphatic regulator *Vegfr3* decreased 129-fold (Fig. 5E). This was likely mediated by 3- to 12-fold reduction in 18 key members of the NF-κB pathway (Fig. 5F) and transcription factors linking immunosuppressive and pro-lymphatic pathways. Notably, regulators of lymphatic proteins (eg, *Lyve1*) such as *Ms4a8a* [44], *Prox1* [45], and *Sox18* [46] were reduced by anti-IL-10R antibody by 2- to 15-fold (Fig. 5G). This treatment also suppressed expression of *Carma1*, an essential scaffold protein for signal transduction of Th2 pathways [47], a stem marker *Sca1* [48], and *Bcl2*, a necessary protein for survival of hematopoietic progenitors [49] (Fig. 5H). This is the first evidence that immunosuppressive IL-10 promotes differentiation of lymphatic progenitors by activation of NF-κB pathway and upregulation of pro-vascular and lymphatic-specific transcription factors.

### TME provides all Th2 ligands for receptor-positive infiltrating myeloid cells

**FIG. 6.**
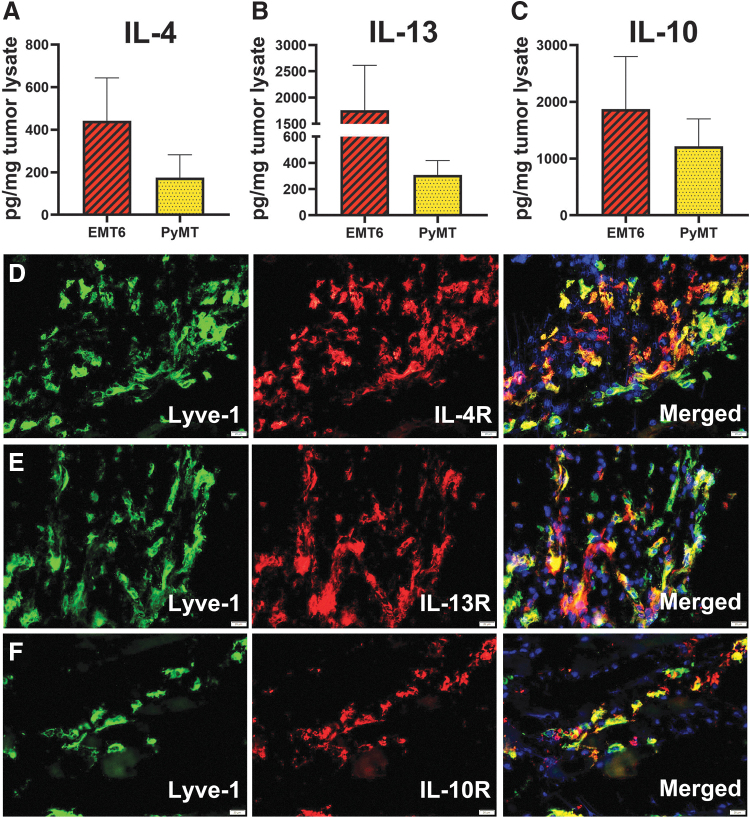
Tumor microenvironment contains IL-4, IL-13, and IL-10, which can activate Th2 receptor-positive myeloid-lymphatic progenitors. BALB/c and C57BL/6 mice were orthotopically implanted in the mammary fat pad with mouse breast cancer lines EMT6 and MMTV-PyMT, respectively. Tumors were harvested when the size reached 500 mm^3^. **(A–C)** Tumor lysates collected from four to five mice were used for measurement of IL-4, IL-13, and IL-10 by ELISA. The mean concentrations ± SD for each cytokine were determined from triplicate readings. **(D–F)** Tumors sections obtained five mice per group were co-stained for a lymphatic marker Lyve-1 and Th2 receptors **(D)** IL-4R, **(E)** IL-13R, or **(F)** IL-10R. Nuclei were visualized by Hoechst's stain. Scale bars are 20 μm. Representative images are shown. All images were acquired at 400 × magnification.

To this point, our data show that M-LECP differentiated in vitro by Th2 cytokines upregulate their own receptors leading to co-induction of M2 and pro-lymphatic phenotypes. To determine the relevance of these data to tumors in vivo, we analyzed expression of Th2 cytokines and receptors in two syngeneic breast tumor models, EMT6 and MMTV-PyMT. We discovered that all three Th2 cytokines were present in tumor lysates of both models (Fig. 6A–C).

Intratumoral expression of IL-4, IL-13, and IL-10 coincided with infiltration of Lyve-1^+^ progenitors that express the corresponding receptors (Fig. 6D–F). These findings suggest that Th2 pathways play an important role in differentiation, and possibly, the function of myeloid-lymphatic progenitors, first, by ensuring their responsiveness through upregulation of Th2 receptors during BM differentiation followed by providing the matching ligands at the tumor site. The TME expression of IL-4/IL-13, absent from the BM, suggests tumor-specific activation of these powerful pathways that drive both immunosuppression and generation of lymphatic vasculature.

## Discussion

The main conclusion from this study is that Th2 cytokines IL-4, IL-13, and IL-10 significantly contribute to pro-lymphatic differentiation of BM-derived myeloid-lymphatic progenitors. All Th2 receptors are upregulated and functional in early myeloid precursors (Figs. 1–3), which is followed by autocrine activation of the IL-10 pathway (Figs. 4 and 5) and reinforced by IL-4 and IL-13 expressed in TME (Fig. 6). These findings underscore co-development of immunosuppressive and pro-lymphatic traits in this subset of tumor-recruited myeloid cells.

At present, pro-oncogenic effects of Th2 cytokines are explained mainly by their induction of immunosuppressive properties in T cells [50] and macrophages [19,51]. However, an increase in Th2 cytokines in cancers and chronically inflamed sites is also associated with generation of blood [29] and lymphatic [52] vessels. For instance, the co-regulated IL-4/IL-13 pathway that shares a type II receptor in hematopoietic cells [53] was shown to induce angiogenic properties in blood-circulating human monocytes [54] and mouse M2 macrophages [26]. This implies that BM-released cells already express IL-4/IL-13 receptors, which is consistent with our data showing their upregulation by CSF-1 and LPS in BM differentiating cells. This is also consistent with evidence for IL-4R and IL-10R expression in human blood-circulating monocytes [55] and myeloid-derived suppressor cells (MDSC) [56], as well as with contribution of both cell types to tumor angiogenesis [57,58].

Th2 pathways are also implicated in lymphangiogenesis. Co-implantation of CSF-1/IL-4-generated M2 macrophages greatly enhanced tumor lymphatic formation and metastasis in lung and breast carcinoma mouse models [52,59], while depletion of M2-TAMs significantly inhibited both processes [60]. In line with our data, BM-derived myeloid cells activated by IL-4/IL-13 upregulated lymphatic-specific markers Lyve-1 and stabilin-1 in several tumor models [33,44]. Lyve-1 and other LEC markers have been consistently detected in M2-TAMs in mouse tumors [33,61] and cancer patients [10].

Transgenic mice overexpressing IL-4 developed inflammatory lymphangiogenesis mediated by influx of CD11b^+^ myeloid cells [62]. Injured IL-10-null mice developed lymphatic insufficiency due to reduced density of M2 macrophages [34]. TLR4 activation by an alternative ligand paclitaxel significantly increased lymphatics and subsequent node metastasis in breast cancer models [63], in line with the reports demonstrating TLR4 prominent role in M-LECP differentiation [9] and induction of Th2 cytokines [64]. These studies taken together with the presented evidence support a novel concept that tumor-induced differentiation of M2 macrophages coincides with the development of lymphatic progenitors that ultimately expand the lymphatic network, thus facilitating malignant spread.

While not broadly recognized, immunosuppression and vascular formation are both associated with M2 macrophage-mediated tissue repair [60]. Th2 cytokines suppress immune activities at the resolving phase of inflammation [20] that is commonly followed by reconstruction of damaged structures. Th2 cytokines induce shift to M2 phenotype that increases macrophage proteolytic activity [65], upregulates scavenger receptors CD163 [36] and CD204 [14] necessary for debris clearance, and confers the ability to suppress immuno-active cells. Both tissue clearance and physical removal of stimulatory immune cells require effective lymphatic drainage to the local nodes [66]. Failure to remove such cells through lymphatics in IL-10-deficient mice led to intense inflammation associated with cytotoxicity [34], which caused tissue damage and was incompatible with tissue repair. Thus, increased lymphatic density might be a functional prerequisite for tissue repair, a process that promotes outgrowth of both malignant and vascular compartments.

## Conclusions

This study demonstrates that immunosuppressive Th2 cytokines play an important role in BM generation of pro-lymphatic progenitors. This new evidence suggests that integration of Th2-targeting strategies into antitumor treatments might concurrently reverse immunosuppression and prevent lymphatic metastasis in cancer patients.

## Supplementary Material

Supplemental data

Supplemental data

Supplemental data

Supplemental data
