# Supplementary material for: Th2 Cytokines IL-4, IL-13, and IL-10 Promote Differentiation of Pro-Lymphatic Progenitors Derived from Bone Marrow Myeloid Precursors
Source: Stem Cells Dev. 2022 Jun 8;31(11-12):322–33. doi: 10.1089/scd.2022.0004 (PMC9232236; doi:10.1089/scd.2022.0004)
Supplement: Supplemental data [file Supp_TableS1.docx]

# **Supplemental Table S1. Antibodies used for flow cytometry, blocking and immunofluorescence**

| Antigen | Vendor | Catalog # | Specie Specificity | Clone Name |
| --- | --- | --- | --- | --- |
| CD11b | BioXCell | BE0007 | Rat anti-mouse (M)^A^ | M1/70 |
| CD115 | BioXCell | BE0213 | Rat anti-mouse (M) | AFS98 |
| CD163 | Spring Bio. | E18682 | Rabbit anti-mouse (P) | N/A^B^ |
| CD204 | Sigma | HPA000272 | Rabbit anti-mouse (P) | N/A |
| CD206 | Thermo | PA546994 | Goat anti-mouse (P) | N/A |
| Collectin-12 | R&D Systems | AF3130 | Goat anti-mouse (P) | N/A |
| IL-4R | Bioss | BS-2458R | Rabbit anti-mouse (P) | N/A |
| IL-10 | BioXcell | BE0049 | Rat anti-mouse (M) | JES5-2A5 |
| IL-10R | Thermo | PA5109852 | Rabbit anti-mouse (P) | N/A |
| IL-10R | BioXCell | BE0050 | Rat anti-mouse (M) | 1B1.3A |
| IL-13R | Sino Bio. | 50088-RP01 | Rabbit anti-mouse (P) | N/A |
| Integrin-α9 | R&D Systems | AF3827 | Goat anti-mouse (P) | N/A |
| Lyve-1 | Angiobio | 11-034 | Rabbit anti-mouse (P) | N/A |
| PD-L1 | BioXCell | BE0101 | Rat anti-mouse (M) | 10F.9G2 |
| Podoplanin | BioXCell | BE0236 | Syrian hamster anti-mouse (M) | 8.1.1 |
| Stabilin-1 | R&D Systems | AF3825 | Sheep anti-human (P) | N/A |
| TLR4 | Biomart | Custom | Rabbit anti-mouse | Peptide 1.2.1 |

^A^M, monoclonal IgG; P, polyclonal IgG

^B^N/A, not applicable
