# Supplementary material for: Th2 Cytokines IL-4, IL-13, and IL-10 Promote Differentiation of Pro-Lymphatic Progenitors Derived from Bone Marrow Myeloid Precursors
Source: Stem Cells Dev. 2022 Jun 8;31(11-12):322–33. doi: 10.1089/scd.2022.0004 (PMC9232236; doi:10.1089/scd.2022.0004)
Supplement: Supplemental data [file Supp_TableS2.docx]

**Supplemental Table S2. RT-qPCR primer sequences**

| **Gene^A^** | **Product Size (bp)** | **Forward Sequence (5' → 3')** | **Reverse Sequence (5' → 3')** |
| --- | --- | --- | --- |
| **Actb** | 153 | GGCTGTATTCCCCTCCATCG | CCAGTTGGTAACAATGCCATGT |
| **Bcl2** | 123 | ATGCCTTTGTGGAACTATATGGC | GGTATGCACCCAGAGTGATGC |
| **Bcl3** | 127 | ACTCACCCCTACTCCATGATATG | GCGGCTATGTTATTCTGGACCA |
| **Carma1** | 90 | CCGGATGAACCTAAAGGGCA | TCGTGCCCCTTGACTTGAAA |
| **CD63** | 138 | AGAGACCAGGTGAAGTCAGAG | AGTCTGTGTAGTTAGAAGCTCCA |
| **CD163** | 108 | GGTGGACACAGAATGGTTCTTC | CCAGGAGCGTTAGTGACAGC |
| **CD204** | 166 | AGTGCTGTCTTCTTTACCAGCA | CTGAAGGGAGGGGCCATTTT |
| **CD206** | 88 | GAGGGAAGCGAGAGATTATGGA | GCCTGATGCCAGGGTAAAGCA |
| **CD209** | 110 | GTTTGTTCTGTGCTGCTGGTT | GCCTTCAACTGGGTCAGTTCT |
| **CD299** | 287 | CTGACAGATGAGCTTACGTCCA | CACAGGCGGAAGAGTTCAGTC |
| **cMaf** | 104 | AGCAAGGAGGAGGTGATCCG | TGTCTCTGCTGCACCCTCTTG |
| **Fizz1** | 100 | CTGCTACTGGGTGTGCTTGT | GCAGTGGTCCAGTCAACGAG |
| **Ifng** | 136 | GAGGTCAACAACCCACAGGT | GGGACAATCTCTTCCCCACC |
| **IL1a** | 251 | CGCTTGAGTCGGCAAAGAAA | CTGATACTGTCACCCGGCTC |
| **IL4** | 101 | GGTCTCAACCCCCAGCTAGT | GCCGATGATCTCTCTCAAGTGAT |
| **IL4R** | 119 | TCTGTGGGCTGTCTGATTTT | GCTATCCAGGAACCACTCAC |
| **IL6** | 131 | TTCTTGGGACTGATGCTGGT | TGTGTAATTAAGCCTCCGAC |
| **IL10** | 277 | CCCAGTCGGCCAGAGC | GCGCCTCAGCCGCAT |
| **IL10R** | 100 | CAAACAGTACGGAAACTCAACCT | GGTGATACAGATCCAGGGTGAAC |
| **IL13** | 116 | CCTGGCTCTTGCTTGCCTT | GGTCTTGTGTGATGTTGCTCA |
| **IL13R** | 119 | CAACAGGATAAGAAAATTGC | AGGGCTAGGCTTCTCACTTT |
| **Itga9** | 207 | AAGTGTCGTGTCCATACCAAC | GGTCTGCTTCGTAGTAGATGTTC |
| **Irak1** | 254 | CTGCTTTGCTGGATGAGCCT | TCACTTTCCACTGGCTGGTT |
| **Irak4** | 243 | GACATCTACAGCTTCGGCGT | CTTTGCAATGTCTGGCCGTC |
| **Lyve1** | 111 | CAGCACACTAGCCTGGTGTTA | CGCCCATGATTCTGCATGTAGA |
| **Mafb** | 178 | TTCGACCTTCTCAAGTTCGACG | TCGAGATGGGTCTTCGGTTCA |
| **Md2** | 122 | ATAAGACTGAGGGGAACCAA | TTCCTTACGCTTCGGCAACT |
| **Mef2b** | 98 | GCAGTCTGTGCAAGGAGG | GGTCCCAGTGACTGGAGAC |
| **Mef2c** | 146 | CCAGATCTCCGCGTTCTT | GGAGTTGCTACGGAAACCA |
| **Ms4a8a** | 95 | CCAAGTGGCCTGTTGTCACT | TTGGGTTTGGTGGTTCTGGG |
| **MyD88** | 188 | CTCGCAGTTTGTTGGATGCC | GGCCACCTGTAAAGGCTTCT |
| **Nemo** | 185 | CCAAGGGAGGTGAGGAGGAA | CTCAGCTGGTTCTTCCAGGG |
| **Nfkb1** | 164 | ACACGAGGCTACAACTCTGC | GGTACCCCCAGAGACCTCAT |
| **Nfkb2** | 136 | CTGCGCTTCTCAGCTTTCCTT | GCTGTCTTGTCCATTCGGG |
| **PDL1** | 192 | TGCCCTTCAGATCACAGACG | TTCTGGATAACCCTCGGCCT |
| **PDL2** | 182 | AGCCTGGAGTGCGATTTTGA | TGCCCGGAATCTCTCACTTG |
| **Prox1** | 147 | GTGGTGCAACACGCAGATG | TGCCACCGTTTTTGTTCATGT |
| **Rel** | 175 | AACAACCGGACATACCCGTC | ACAAAGGTCTGCGTTCTGGT |
| **Rela** | 75 | AGTTCATGTGGATGAGGCCG | GCTACACGGGACCAGGAACAG |
| **Relb** | 142 | CCGTACCTGGTCATCACAGAG | CAGTCTCGAAGCTCGATGGC |
| **Sca1** | 185 | CTCCCAAGAAACGTGAGATCC | CCATTCCTTGTAAACCATGCTCC |
| **Sox18** | 187 | CGCAGGTCTCTACTATGGCAC | TAGTGGCATCCGGTCGAGT |
| **Stab1** | 146 | GGCAGACGGTACGGTCTAAAC | AGCGGCAGTCCAGAAGTATCT |
| **Stat3** | 142 | TACCTCTACCCCGACATTCCC | CATCAATGAATGGTGTCACACAGA |
| **Stat5** | 113 | CGCCAGATGCAAGTGTTGTAT | TCCTGGGGATTATCCAAGTCAAT |
| **Stat6** | 189 | TGGTCCTGGTCCAAGTGAGG | ATTTCCACCAGGCTTTCACA |
| **Tab1** | 102 | TCCAACCGCAGCTACTCTG | CCCGTACAGGAAGCAGTTATTTT |
| **Tank** | 127 | AGACATAGTCTGCGAAGGAACG | ATGCTCTATTGAGTTGCTCACC |
| **Ticam1** | 109 | GAGGCAGGACTGTGTGATCC | GGAGTGTTCATCCAGCCACA |
| **Tlr2** | 230 | GCAAACGCTGTTCTGCTCAG | AGGCGTCTCCCTCTATTGTATT |
| **Tlr4** | 128 | ATGGCATGGCTTACACCACC | GAGGCCAATTTTGTCTCCACA |
| **Tlr6** | 138 | TGAGCCAAGACAGAAAACCCA | GGGACATGAGTAAGGTTCCTGTT |
| **Traf6** | 146 | AGTGAAAGATGACAGCGTGA | TCCCGTAAAGCCATCAAGCA |
| **Tnfa** | 102 | CCACCACGCTCTTCTGTCTAC | AGGGTCTGGGCCATAGAACT |
| **Vegfa** | 133 | AGCAACATCACCATGCAGAT | TCACAGTGATTTTCTGGCTTTG |
| **Vegfd** | 62 | GAGGACTGGAAGCTGTGGCG | GCGTGAGTCCATACTGGCAAGA |
| **Vegfr1** | 162 | CTCAGACAAGTCAAACCTGGAG | GGGAACTTCATCTGGGTCCATAA |
| **Vegfr2** | 132 | TTTGGCAAATACAACCCTTCAGA | GCAGAAGATACTGTCACCACC |
| **Vegfr3** | 194 | CGGGCTACCTGTCCATCATC | TGTCACAGCTGCTGCCTTTA |
| **Ym1** | 112 | GTACCCTGGGTCTCGAGGAA | CCTTGGAATGTCTTTCTCCACAG |

^A^ Primers were designed based on mouse CDS of targets found in NCBI database. All primers were validated using mouse universal cDNA.
