# Supplementary material for: Th2 Cytokines IL-4, IL-13, and IL-10 Promote Differentiation of Pro-Lymphatic Progenitors Derived from Bone Marrow Myeloid Precursors
Source: Stem Cells Dev. 2022 Jun 8;31(11-12):322–33. doi: 10.1089/scd.2022.0004 (PMC9232236; doi:10.1089/scd.2022.0004)
Supplement: Supplemental data [file Supp_TableS3.docx]

**Supplemental Table S3. Effects of IL-4, IL-13, and IL-10 on cell density and diameter of CSF-1 primed precursors**

| Treatment | # Cells (10^6^) per dish^A^ | P-value vs. CSF-1 treated cells | Diameter (µm)^A^ | P-value vs. e*x vivo* cells | P-value vs. CSF-1 treated cells |
| --- | --- | --- | --- | --- | --- |
| None (*ex vivo*) | N/A^B^ | N/A | 7.58 ± 0.29^C^ | N/A | <0.05 |
| CSF-1 | 0.42 ± 0.03 | N/A | 9.76 ± 0.46 | <0.05 | N/A |
| IL-4 | N/D^D^ | N/A | N/D | N/A | N/A |
| CSF-1 + IL-4 | 1.00 ± 0.14 | <0.05 | 12.01 ± 0.24 | <0.05 | <0.05 |
| IL-13 | N/D | N/A | N/D | N/A | N/A |
| CSF-1 + IL-13 | 1.08 ± 0.12 | <0.05 | 11.78 ± 0.53 | <0.05 | <0.05 |
| IL-10 | N/D | N/A | N/D | N/A | N/A |
| CSF-1 + IL-10 | 0.84 ± 0.23 | <0.05 | 11.45 ±0.05 | <0.05 | <0.05 |

^A^Parameters recorded on day 6 of differentiation

^B^N/A, not applicable

^C^Diameter recorded on the day of cell isolation

^D^ N/D, not done; analyses were not performed due to low cell survival
