# Supplementary material for: Th2 Cytokines IL-4, IL-13, and IL-10 Promote Differentiation of Pro-Lymphatic Progenitors Derived from Bone Marrow Myeloid Precursors
Source: Stem Cells Dev. 2022 Jun 8;31(11-12):322–33. doi: 10.1089/scd.2022.0004 (PMC9232236; doi:10.1089/scd.2022.0004)
Supplement: Supplemental data [file Supp_FigS1-S3.docx]

**Supplemental Figure S1. Morphology of BM cells differentiated with CSF-1 or IL-4 alone or combined treatment.** BM cells were differentiated for 6 days with **(A)** CSF-1 (10 ng/ml), **(B)** IL-4 (10 ng/ml) or **(C)** for 3 days with CSF-1 followed by a 3-day treatment with IL-4 (6 days total). Representative images of BM cells on days 4 through 6 are presented. Scale bars are 50 µm. All images were acquired at 200X magnification.

**Supplemental Figure S2. Morphology of BM cells differentiated with CSF-1 or IL-10 alone or combined treatment.** BM cells were differentiated for 6 days with **(A)** CSF-1 (10 ng/ml), **(B)** IL-10 (10 ng/ml) or **(C)** for 3 days with CSF-1 followed by a 3-day treatment with IL-10 (6 days total). Representative images of BM cells on days 4 through 6 are presented. Scale bars are 50 µm. All images were acquired at 200X magnification.

**Supplemental Figure S3. Morphology of BM cells differentiated with CSF-1 or IL-13 alone or combined treatment.** BM cells were differentiated for 6 days with **(A)** CSF-1 (10 ng/ml), **(B)** IL-13 (10 ng/ml) or **(C)** for 3 days with CSF-1 followed by a 3-day treatment with IL-13 (6 days total). Representative images of BM cells on days 4 through 6 are presented. Scale bars are 50 µm. All images were acquired at 200X magnification.
